# A multiplex urinary immunoassay for bladder cancer detection: analysis of a Japanese cohort

**DOI:** 10.1186/s12967-016-1043-1

**Published:** 2016-10-07

**Authors:** Steve Goodison, Osamu Ogawa, Yoshiyuki Matsui, Takashi Kobayashi, Makito Miyake, Sayuri Ohnishi, Kiyohide Fujimoto, Yunfeng Dai, Yoshiko Shimizu, Kazue Tsukikawa, Hideki Furuya, Charles J. Rosser

**Affiliations:** 1 Department of Health Sciences Research, Mayo Clinic, Jacksonville, FL USA; 2 Mayo Clinic Cancer Center, Rochester, MN USA; 3 Department of Urology, Kyoto University, Kyoto, Japan; 4 Department of Urology, Nara Medical University, Nara, Japan; 5 Department of Biostatistics, The University of Florida, Gainesville, FL USA; 6 Clinical and Translational Research Program, University of Hawaii Cancer Center, 701 Ilalo St, Rm 327, Honolulu, HI 96813 USA

**Keywords:** Biomarkers, Bladder cancer, Multiplex, Protein, Urine

## Abstract

**Background:**

Bladder cancer (BCa) is among the most commonly diagnosed malignancies worldwide, and due the high rate of post-operative disease recurrence, it is one of the most prevalent in many countries. The development of non-invasive molecular assays that can accurately detect and monitor BCa would be a major advance, benefiting both patients and healthcare systems. We have previously identified a urinary protein biomarker panel that is being developed for application in at-risk patient cohorts. Here, we investigated the potential utility of the multiplex assay in a Japanese cohort.

**Methods:**

The Japanese study cohort collected from urology clinics at two institutions was comprised of a total of 288 subjects. The protein biomarker panel (IL8, MMP9, MMP10, ANG, APOE, SDC1, A1AT, PAI1, CA9, VEGFA) was monitored in voided urine samples collected prior to cystoscopy using a custom multiplex ELISA assay. The diagnostic performance of the biomarker panel was assessed using receiver operator curves, predictive modeling and descriptive statistics.

**Results:**

Urinary biomarker concentrations were significantly elevated in cases versus controls, and in cases with high-grade and muscle-invasive tumors. The AUC for the 10-biomarker assay was 0.892 (95 % confidence interval 0.850–0.934), with an overall diagnostic sensitivity specificity of 0.85 and 0.81, respectively. A predictive model trained on the larger institutional cohort correctly identified 99 % of the cases from the second institution.

**Conclusions:**

Urinary levels of a 10-biomarker panel enabled discrimination of patients with BCa. The multiplex urinary diagnostic assay has the potential to be developed for the non-invasive detection of BCa in at-risk Japanese patients.

## Background

Bladder cancer (BCa) is a major burden for both patients and health care systems worldwide [1]. At initial presentation, the majority of cases are non-muscle invasive tumors, which are not immediately life-threatening. However, more than 70 % of patients with operable BCa will have a recurrence during the first 2 years after diagnosis and the subsequent lesions can progress to being muscle invasive and metastatic [2]. Thus, once treated, BCa patients are under continual surveillance with routine cystoscopy examinations for early detection of new BCa development. Due to the prolonged natural history of BCa, plus the prolonged and invasive nature of follow-up and treatment strategies, it is one of the most expensive malignancies to manage on a per-patient basis [3]. Although Japan and other Asian countries have a somewhat lower incidence of BCa than Europe and the Americas, the clinico-pathological characteristics of the disease are similar. In Japan, around 12,000 patients are newly diagnosed and 5000 patients die from the disease annually. While the overall incidence trend has been relatively stable over the past decade [4, 5], because the onset is most often seen in elderly patients, it is becoming a major social issue in an aging Japanese society.

In a series of previous studies, we have identified panels of protein biomarkers that are significantly associated with BCa [6, 7]. Through several studies [8–10] we have refined the target composition, and the immunoassay approach [11], in order to develop a viable non-invasive assay for BCa detection. In this study, we tested the potential utility of the multiplex immunoassay for the detection of BCa in a Japanese cohort. Evaluation of 288 subjects obtained from two independent institutes confirmed a significant association of the tested biomarkers with the presence of BCa. The multiplex assay achieved a strong overall diagnostic performance achieving 85 % sensitivity and 81 % specificity (AUC 0.892). This retrospective phase II study confirms the potential of using urinary protein biomarker signatures for the non-invasive detection of BCa, and suggests that the described multiplex immunoassay could aid in Japanese urology patient management.

## Methods

### Patients and specimen processing

Under Western Institutional Review Board approval (IRB #Rosser 2014-1), previously collected and banked voided urine samples shipped to University of Hawaii Cancer Center for analysis. Originally, voided urine samples were collected prior to cystoscopy, and stored frozen until analysis. The study cohort (Table 1) was comprised of subjects from two independent institutions; the Kyoto cohort consisted of 80 subjects with newly diagnosed BCa, and 67 subjects with no active BCa (controls) including subjects with voiding symptoms, renal or prostate cancer, urinary tract infections or urolithiasis. The Nara cohort consisted of 131 subjects with newly diagnosed BCa. For the bladder cancer case group, histological confirmation of urothelial carcinoma, including grade and stage was defined from excised tissue. Frozen aliquots of urine samples were thawed and protein content was measured using a Pierce 660-nm Protein Assay Kit (Thermo Fisher Scientific Inc., Waltham, MA, USA). The concentration of urinary creatinine was measured using a commercially available enzymatic assay (Cat#KGE005 R&D Systems Inc., Minneapolis, MN, USA) according to the manufacturer’s instructions. Normalization to creatinine is the standard means of expressing analytes in urine to minimize the impact of patient hydration status [12].

**Table 1:** Demographic and clinical-pathologic characteristics of study cohorts

	Bladder cancer (n = 211)	control (n = 67)
Median age (range, years)	75 (22–95)	70 (30–90)
Male:female ratio	183/28	53/14
Stage		n/a
NMIBC	170 (80.6 %)	
MIBC	41 (19.4 %)	
Grade		n/a
Low	87 (41.2 %)	
High	124 (58.8 %)	
Clinical stage		n/a
Tis high-grade	7 (3.3 %)	
Ta low-grade	80 (37.9 %)	
Ta high-grade	29 (13.7 %)	
T1 low-grade	7 (3.3 %)	
T1 high-grade	47 (22.3 %)	
≥T2 high-grade	41 (19.4 %)	

### Multiplex immunoassay

The concentrations of the 10 proteins (IL8, MMP9, MMP10, ANG, APOE, SDC1, A1AT, PAI1, CA9 and VEGFA) were monitored using a custom multiplex immunoassay using MULTI-ARRAY® technology (Meso Scale Diagnostics, LLC, Rockville, MD). The multiplex assay, using MULTI-SPOT® plates, is based on a proprietary combination of electrochemiluminescence detection and patterned arrays [13–15]. Final monoclonal antibody pairs (capture and detection) were selected based on sensitivity, specificity, physical properties, and recognition of native protein, as described previously [11]. In order to ensure detection across the range of protein concentrations, 7 of the 10 assays (IL8, MMP9, MMP10, APOE, PAI1, CA9 and VEGFA) were multiplexed in one well, and the other 3 assays (ANG, SDC1, A1AT) in a separate well. Urine samples were diluted fourfold for the 7-plex assay and 200-fold for the 3-plex assay. A seven point standard curve across the 4 log dynamic range of the assays was included in the current assay design. Urine samples were handled on ice and diluted with MSD Assay Diluent 37 [11]. Samples and standards (50 μl) were added to the plate and incubated for 2 h at RT. After washing, SULFO-TAG conjugated detection antibody mix (25 μl) was added and incubated for 2 h at RT. After washing, the ensuing immunoassay complex was incubated with MSD Read Buffer (150 μl) and electrochemiluminescence measured on the QuickPlex® SQ 120 (MSD) instrument. Standard curves were constructed using MSD Discovery Workbench® 4.0, which allows for the selection of multiple non-linear and linear equations to fit the standard curve. Optimal curve fits were determined by visual graph evaluation and comparison of akaike’s information criteria (AIC) values [16].

### Data analysis

Wilcoxon rank sum tests were used to determine the association between each biomarker and BCa. Nonparametric receiver operating characteristic (ROC) curves were generated to plot assay sensitivity against the false-positive rate (1-specificity). The relative ability of each biomarker to indicate BCa was evaluated by calculating the area under the curve (AUC), and AUCs were compared by Chi-square test. The sensitivity and specificity of each biomarker and their combinations were estimated at the optimal cutoff value defined by the Youden index [17]. Furthermore, the formula for calculating the combined score of prediction was −3.3600 + (0.4889)*normalized IL8 + (−2.9371)*normalized MMP9 + (0.2112)*normalized A1AT + (−0.0470)*normalized ANG + (−0.5617)*normalized VEGFA + (1.7788)*normalized CA9 + (−0.1412)*normalized MMP10 + (−3.0268)*normalized APOE + (47.5054)*normalized PAI1 + (7.4465)*normalized SDC1 > 0.80385). To assess the independent association between biomarkers and BCa, we used logistic regression analysis with BCa status (yes vs. no) as the response variable and biomarker concentrations as explanatory variables. Multiple-level logistic regression was used to evaluate biomarker association with tumor grade and muscle-invasive disease. The all-subset method was used to evaluate the predictive value of each possible combination of biomarkers, and the Bayesian information criterion (BIC) was used to compare models. The BIC, a widely used criterion in model selection, balances the model likelihood and the number of biomarkers included in the model [18]. The Bootstrap method (using 1000 Bootstrap samples) was used [19] to select the most efficient and stable predictive model. Sensitivity, specificity, positive predictive value (PPV), and negative predictive value (NPV) were calculated from ROC data. Statistical significance in this study was set at P < 0.05 and all reported P values were 2-sided. All analyses were performed using SAS software version 9.4.

## Results

### Patient characteristics

Clinical, pathologic and demographic characteristics of the 288 subjects (183 BCa, 96 benign controls, and 41 healthy volunteers) comprising the study cohort are listed in Table 1. Of the 211 bladder cancer cases, 170 were classified as non-muscle invasive bladder cancer (NMIBC; stages Ta, Tis, T1), and 41 were muscle invasive bladder cancer (MIBC; stage ≥ T2). Eighty-seven cases were reported as low-grade carcinoma and 98 cases as high-grade. Gender distribution (BCa incidence is higher in men), and slightly older median age for patients in the BCa group reflect typical BCa incidence statistics [1].

### Urinary biomarker levels and assay performance

**Fig. 1:**
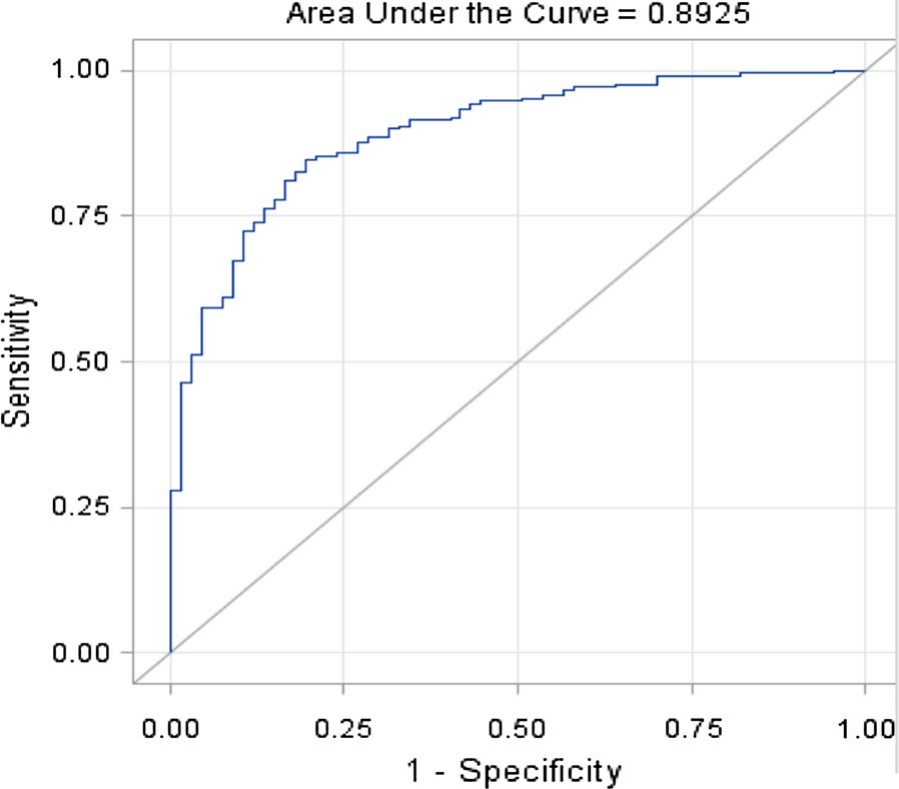
Diagnostic performance of a multiplex protein biomarker assay. ROC was plotted to describe performance characteristics in a 288 subject cohort. Area under the curve (AUC) 0.8925

Biomarker concentration data were normalized to urinary creatinine [12] to adjust for urine volume variations and to be consistent with our previous studies [8–11]. Urinary concentrations of all 10 biomarkers were significantly elevated in patients with bladder cancer compared with controls (Table 2). The concentrations of 9 of the 10 biomarkers (except SDC1) were also significantly elevated in patients with high-grade BCa relative to low-grade BCa, and MIBC relative to NMIBC (Table 2). The diagnostic performance of each individual biomarker was analyzed using nonparametric ROC analyses [20]. As observed previously, many of the individual biomarkers achieved respectable diagnostic performance values, with SDC1 and SERPINA1 both recording AUC values >0.80. Table 3 provides AUC and corresponding sensitivity, specificity, PPV, and NPV values for all biomarkers tested. The optimal test was derived by combination of the 10 biomarkers into a diagnostic panel. In line with our previous multi-institutional studies with cohorts from the USA and Europe [9, 10], the 10-biomarker assay achieved an AUC of 0.8925 in the Japanese cohort (Fig. 1), with an overall diagnostic sensitivity of 0.895 and specificity of 0.806 (Table 3). As expected, sensitivity and specificity values of the combined assay (Table 4) were higher for high-grade (90.3/85.1 %) and for MIBC bladder cancer (85.4/95.5 %), but the overall performance was comparable. Although data was incomplete, voided urine cytology data indicated sensitivity values of ~45 % for those subjects tested in this cohort.

**Table 2:** Mean urinary (±SD) concentrations of ten biomarkers assessed by ELISA in cohort of 288 subjects

Biomarker	Total bladder cancer	Low-grade bladder cancer	High-grade bladder cancer	NMIBC	MIBC	Controls
	(75.9 %)	(41.2 %)	(58.8 %)	(80.6 %)	(19.4 %)	(24.1 %)
	*n* = 211	*n* = 87	*n* = 124	*n* = 170	*n* = 41	*n* = 67
IL8 (pg/mL)^abc^	1.224 ± 1.387	0.763 ± 0.824	1.547 ± 1.598	1.020 ± 1.177	2.070 ± 1.826	0.566 ± 0.464
MMP9 (ng/mL)^abc^	0.485 ± 0.536	0.311 ± 0.261	0.607 ± 0.638	0.411 ± 0.434	0.792 ± 0.770	0.319 ± 0.366
SERPINA1 (ng/mL)^abc^	2.648 ± 2.538	1.891 ± 1.594	3.180 ± 2.920	2.312 ± 2.147	4.041 ± 3.444	1.061 ± 0.711
ANG (pg/mL)^abc^	2.133 ± 1.181	1.812 ± 0.928	2.358 ± 1.286	1.979 ± 1.037	2.770 ± 1.504	1.190 ± 0.543
VEGF (pg/mL)^abc^	1.852 ± 1.021	1.570 ± 0.610	2.050 ± 1.193	1.731 ± 0.897	2.352 ± 1.322	1.386 ± 0.395
CA9 (pg/mL)^abc^	0.770 ± 0.971	0.577 ± 0.480	0.905 ± 1.185	0.668 ± 0.805	1.190 ± 1.411	0.357 ± 0.242
MMP10 (pg/mL)^abc^	0.989 ± 1.625	0.626 ± 0.422	1.243 ± 2.055	0.814 ± 1.346	1.713 ± 2.353	0.517 ± 0.492
APOE (pg/mL)^abc^	0.639 ± 0.427	0.491 ± 0.307	0.744 ± 0.468	0.571 ± 0.373	0.923 ± 0.518	0.420 ± 0.177
SERPINE1 (ng/mL)^abc^	0.156 ± 0.181	0.096 ± 0.080	0.198 ± 0.217	0.125 ± 0.134	0.284 ± 0.273	0.056 ± 0.030
SDC1 (pg/mL)^a^	0.489 ± 0.140	0.468 ± 0.127	0.504 ± 0.147	0.478 ± 0.129	0.534 ± 0.172	0.331 ± 0.121

*NMIBC* non-muscle invasive bladder cancer, *MIBC* muscle invasive bladder cancer

^a^ *P* < 0.05 comparing total bladder cancer to total controls

^b^ P < 0.05 comparing low-grade bladder cancer to high-grade bladder cancer

^c^ P < 0.05 comparing NMIBC to MIBC

**Table 3:** Individual and combined biomarker performance data for bladder cancer detection

Biomarker	AUC	95 % CI	No of correctly predicted events	No of correctly predicted nonevents	No of nonevents predicted as events	No of events Predicted as nonevents	Sensitivity	Specificity	PPV	NPV
IL8	0.7039	0.6332–0.7746	151	43	24	60	0.716	0.642	0.863	0.417
MMP9	0.6315	0.5541–0.7090	104	51	16	107	0.493	0.761	0.867	0.323
SERPINA1	0.8027	0.7432–0.8622	163	49	18	48	0.773	0.731	0.901	0.505
ANG	0.7835	0.7241–0.8430	155	49	18	56	0.735	0.731	0.896	0.467
VEGFA	0.6488	0.5774–0.7202	89	54	13	122	0.422	0.806	0.873	0.307
CA9	0.7202	0.6550–0.7855	100	59	8	111	0.474	0.881	0.926	0.347
MMP10	0.6214	0.5433–0.6996	175	29	38	36	0.829	0.433	0.822	0.446
APOE	0.6298	0.5614–0.6983	83	63	4	128	0.393	0.940	0.954	0.330
SERPINE1	0.7989	0.7406–0.8571	141	54	13	70	0.668	0.806	0.916	0.435
SDC1	0.8161	0.7602–0.8719	145	58	9	66	0.687	0.866	0.942	0.468
10-Biomarker combination	0.8925	0.8507–0.9343	179	54	13	32	0.848	0.806	0.932	0.628

*AUC* area under the curve, CI confidence interval, *PPV* positive predictive value, *NPV* negative predictive value

**Table 4:** Summary of diagnostic sensitivity of 10-biomarker panel and voided urinary cytology in patients with bladder cancer

	No of bladder cancer cases predicted by biomarker assay	AUC	Sensitivity (%)	Specificity (%)
Overall	179 of 211	0.895	84.8	80.6
Low-grade tumors	63 of 87	0.849	72.4	82.1
High-grade tumors	112 of 124	0.932	90.3	85.1
NMIBC	139 of 170	0.878	81.8	80.6
MIBC	35 of 41	0.961	85.4	95.5

*NMIBC* non-muscle invasive bladder cancer, *MIBC* muscle invasive bladder cancer, *AUC* Area under ROC curve

The overall classification data was obtained through analysis of data from the total cohort, but given that subjects were combined from two independent institutes, we could also investigate the ability of a predictive model derived from one institute to identify cases in the second institute. The Kyoto cohort (147 subjects) had a balance of 80 cases and 67 controls, so this was used to build a predictive model. The best logistic regression model for the 10-biomarker panel correctly identified 56 of the 80 Kyoto cases. Although this was less accurate than the overall classification analyses described above, when applied to the Nara cohort the Kyoto-derived predictive model correctly identified 130 of the 131 Nara cases as harboring BCa.

## Discussion

Bladder cancer is among the most common worldwide malignancies, and due to the high recurrence rate, it is also one of the most burdensome for both patients and healthcare systems. Current guidelines for BCa diagnosis recommend cystoscopy coupled with voided urine cytology (VUC), but cystoscopy is an invasive procedure associated with discomfort and anxiety, infection and trauma [21, 22]. Non-invasive VUC is used routinely as an adjunct to cystoscopy, but the subjective assay can be impaired by inter-observer variability and has poor sensitivity, especially for low-grade and low-stage tumors [23, 24]. Urine-based, quantitative assays that can non-invasively detect BCa would improve the rapid diagnosis of BCa and help to avoid unnecessary invasive cystoscopy and biopsy procedures, however, while a number of such assays have been proposed, to date, such assays have lacked adequate accuracy to replace VUC, or to support or guide cystoscopy. The lack of accuracy of current urinary tests may be due to the fact that they typically rely on measuring a single biomarker. A shift from single biomarker assays to multiplex molecular signatures that reflect the molecular pathways of BCa provides an opportunity to develop assays with clinical utility across the breadth of disease states. The advent of high-throughput technologies has facilitated the discovery of more complex molecular signatures with diagnostic or prognostic potential, and a number of gene expression signature assays are now being incorporated into clinical practice [25, 26].

Through proteomic profiling of naturally micturated urine, we were able to identify a set of urinary proteins that were associated with the presence of BCa [6, 7]. One advantage of profiling the urine component directly is the ability to compare samples collected from subjects with non-malignant conditions, a situation that is not feasible using surgically excised solid tissue samples. The candidate biomarkers were refined through independent validation studies [27, 28], and a 10-biomarker panel was established for development. The diagnostic performance of the 10-protein assay was then tested in two independent cohorts, one [9] comprised of 127 subjects (diagnostic sensitivity of 92 %, specificity of 97 %), and one [10] comprised of 308 patients obtained from multiple institutions in the USA and Europe (sensitivity 74 %, specificity 90 %). Taken together, these studies illustrate the reproducibility of the diagnostic protein panel in terms of a robust biomarker panel that achieved similar performance data across multiple, independent cohorts. Based on these results, we investigated the feasibility of developing a multiplex assay that could accurately and simultaneously monitor the diagnostic biomarkers in an efficient format for potential clinical application. A custom-designed multiplex assay using MULTI-ARRAY® technology (meso scale diagnostics, LLC) was constructed, and the analytical performance was compared with data obtained from individual ELISA assays directed at each of the same 10 urinary proteins. The multiplex assay achieved excellent concordance, and improved detection range and technical sensitivity [11]. The diagnostic performance of the assay was confirmed in an independent cohort of 200 subjects (sensitivity 85 %, specificity 81 %). The integration of multiplex molecular signatures into a single assay is beneficial for clinical translation through reduced sample volume, decreased processing time, low cost analysis and low reagent consumption, and the MULTI-ARRAY® assay can be readily implemented into a CLIA certified laboratory setting using existing instrumentation and skillsets.

Here, we extended the evaluation of the multiplex BCa diagnostic assay to a phase II, multicenter study with a cohort comprised of 288 Japanese subjects. For overall classification, the multiplex assay achieved a sensitivity of 84.8 % and specificity of 80.6 % (AUC of 0.892). Biomarker expression and assay performance increased with increasing tumor stage and grade. The results in the Japanese cohort were very much in line with our previous studies evaluating cohorts from the USA and Europe [9–11], but as we refine and optimize the technical aspects of the assay and analyze additional samples, we expect to be able to further improve assay performance. While subtle differences in composition of optimal urinary diagnostic protein panels may exist in cohorts comprised of different ethnic populations, given the translation of an assay optimized in diverse cohorts to a Japanese population, these data are very encouraging, and further validate the concept that a multiplex panel of urinary protein biomarkers can assist the noninvasive diagnosis of BCa.

We recognize that the study has a number of limitations. The cohort was comprised of >50 % cases, but disease prevalence is typically considerably lower in routine urologic practice. While it was important to initially test enough cases to achieve statistical significance, it will be necessary to perform additional studies that reflect urology clinic presentation to assess predictive value of the assay. It will also be necessary to include more diverse controls that may be under-represented in our study cohort. To address these issues, we have launched a multi-institute, prospective clinical trial, which will assess the multiplex diagnostic assay in subjects with gross hematuria, microscopic hematuria and history of BCa on tumor surveillance. Such a study would minimize selection bias, better represent urological disease prevalence, and evaluate potential confounding comorbidities in the study population. Furthermore, in this study, we focused only on subjects with primary BCa, however, we have previously reported that the multiplex assay is also accurate for the detection of recurrent BCa (sensitivity 79 %, specificity 88 %), outperforming the Urovysion cytogenetic assay and VUC [29]. The inclusion of Japanese patients on routine surveillance after primary BCa treatment will be an additional goal and will enable evaluation of potential prognostic utility of the assay. Finally, we have initiated the development of BCa diagnostic nomograms that incorporate biomarker data with relevant clinical information (e.g., age, sex, race, and tobacco history) in US cohort studies, and this can also be extended to future Japanese cohort studies.

## Conclusion

Bladder cancer is a common neoplastic disease with a high rate of recurrence and progression, and the recurrence phenomenon makes it one of the most prevalent cancers worldwide. The development of robust non-invasive, urine-based assay for the detection of BCa is clinically urgent. In this study, we have been able to successfully translate a multiplex diagnostic assay derived from diverse cohort studies to the analysis of a Japanese population. The diagnostic assay achieved encouraging performance values and will be the focus of ongoing studies to investigate the potential added value of the multiplex assay if integrated into clinical decision making.

## Abbreviations

BCa: bladder cancer
ROC: receiver operator curves
AUC: area under the curve
IL8: interleukin 8
MMP: matrix metallopeptidase
ANG: angiogenin
APOE: apolipoprotein E
SDC1: syndecan 1
A1AT: alpha-1 antitrypsin
PAI1: plasminogen activator inhibitor-1
CA9: carbonic anhydrase 9
VEGFA: vascular endothelial growth factor A
MSD: meso scale diagnostics
AIC: akaike’s information criteria
BIC: bayesian information criterion
PPV: positive predictive value
NPV: negative predictive value
NMIBC: non-muscle invasive bladder cancer
MIBC: muscle invasive bladder cancer
VUC: voided urine cytology
